# EPHA4 is overexpressed but not functionally active in Sézary syndrome

**DOI:** 10.18632/oncotarget.5573

**Published:** 2015-09-10

**Authors:** Liesbeth Hameetman, Leslie van der Fits, Willem H. Zoutman, Jacoba J. Out-Luiting, Gregg Siegal, Iwan J.P. de Esch, Maarten H. Vermeer, Cornelis P. Tensen

**Affiliations:** ^1^ Department of Dermatology, Leiden University Medical Center, Leiden, The Netherlands; ^2^ Leiden Institute of Chemistry, Leiden University, Leiden, The Netherlands; ^3^ Leiden/Amsterdam Center for Drug Research (LACDR), Division of Medicinal Chemistry, Faculty of Sciences, VU University Amsterdam, Amsterdam, The Netherlands

**Keywords:** cutaneous T-cell lymphoma, Sézary syndrome, EPHA4

## Abstract

EPHA4 belongs to the largest subfamily of receptor tyrosine kinases. In addition to its function during development, overexpression of *EPHA4* in tumors has been correlated with increased proliferation, migration and poor survival. Several genome-wide transcription profiling studies have demonstrated high *EPHA4* expression in Sézary syndrome (SS), a leukemic variant of cutaneous CD4+ T-cell lymphoma (CTCL) with an aggressive clinical course and poor prognosis. In this study we set out to explore the functional role of EPHA4 in SS. Both high EPHA4 mRNA and protein expression was found in circulating SS-cells of patients compared to healthy CD4+ T-cells. However, using a phosphospecific EPHA4 antibody, phosphorylation of the EPHA4 kinase domain was not detected in either circulating or skin residing SS cells. Moreover, treatment with the phosphatase inhibitor pervanadate did not result in detectable phosphorylation of the EPHA4 kinase domain, in either SS cells or in healthy CD4+ T-cells. Thus, the results from our study confirm high EPHA4 expression in SS cells both on the mRNA and protein levels, making EPHA4 a good diagnostic marker. However, the overexpressed EPHA4 does not appear to be functionally active and its overexpression might be secondary to other oncogenic drivers in SS, like STAT3 and TWIST1.

## INTRODUCTION

Sézary syndrome (SS) is a rare leukemic variant of cutaneous T-cell lymphoma (CTCL) that is characterized by erythroderma, generalized lymphadenopathy, and the presence of neoplastic CD4+ skin-homing memory T-cells (SS cells) in the skin, lymph nodes, and peripheral blood. [1–3]. Diagnosis is based on the clinical presentation, presence of SS cells in the skin, detection of clonally rearranged T-cell receptor (TCR) in both skin and peripheral blood, as well as on one or more aberrancies in the peripheral blood, such as an absolute count of > 1000 SS cells mm^3^, a CD4/CD8 ratio of > 10, and loss of T-cell markers such as CD2, CD3 and/or CD5 [2, 4]. In early stages of disease, differentiation between SS and benign erythroderma (BE) secondary to atopic dermatitis, chronic dermatitis, or adverse drug reactions, can be very difficult. SS often has an aggressive clinical course and poor prognosis (estimated 5-year survival < 25%) and until now, the results of various treatments for SS are generally disappointing.

Studies investigating the pathogenesis of SS demonstrated aberrations at both genomic [5, 6] and expression level, which have implicated pathogenic involvement of signaling pathways such as STAT [7–9] and NOTCH1 [10]. Three independent genome-wide gene expression studies all found high mRNA expression of the receptor tyrosine kinase (RTK), *EPHA4*, in SS but not in other CTCLs or BEs [11–13]. This suggests that EPHA4 might be a specific diagnostic marker and potentially, an attractive target for therapeutic intervention.

EPH receptors are the largest subfamily of RTKs [14, 15]. In the human genome, fourteen EPH receptors have been identified, which can be divided in two classes, EPHA and EPHB receptors respectively, based on sequence similarities and ligand-binding properties. EPH receptors are activated by their cell surface-anchored ligands, so-called Ephrins. Ephrins are attached to the cell membrane either *via* a glycosylphosphatidylinositol (GPI) anchor linkage (A class) or a transmembrane sequence (B class). Since initial identification as important mediators during development of the central nerve system [16], EPH/Ephrin signaling was shown to be involved in many different cell-cell interactions and cellular processes [17–19], with EPHA4 as an essential regulator in the correct guidance of spinal cord axons during development[16, 20, 21]. Deregulation of EPH/Ephrin signaling has been demonstrated in a variety of diseases, including diabetes, neurodegenerative disorders and cancer [19, 22].

In addition to Sézary syndrome, EPHA4 has also been found to be overexpressed in several different types of solid tumors, such as prostate, pancreatic, liver and gastric cancer [23–26]. In gastric cancer EPHA4 overexpression has been correlated with poor prognosis [24] and depletion of *EPHA via* siRNA resulted in decreased proliferation and lower migration [23, 27]. In contrast, EPHA4 has recently been described as an inhibitor of cell migration and invasion in lung cancer [28].

The aim of the current study was to explore the functional role of EPHA4 as a RTK in SS cells. We set out to confirm the previously observed high expression of both mRNA and protein and investigate if EPHA4 is active, by looking at phosphorylation of the kinase domain in both tumor cells in the peripheral blood and skin. Moreover, we investigated EPHA4 activation *in vitro* in SS cells and healthy CD4+ T-cells.

## RESULTS

### EPHA4 is overexpressed in primary SS cells but not in SS cell lines

**Figure 1:**
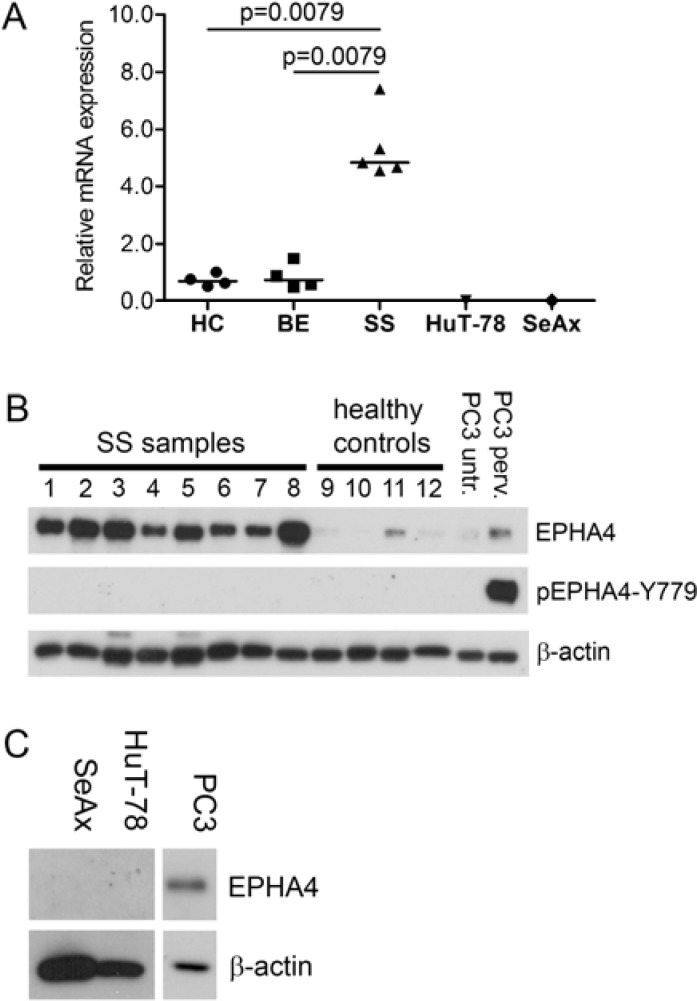
EPHA4 and phosphorylated EPHA4 expression in SS **A.** RNA was extracted from CD4+ T-cells isolated from peripheral blood from five SS patients, four benign erythroderma samples (BE) and the SS cell lines SeAx and HuT-78. EPHA4 expression was assayed by qPCR and normalized for expression of the stably expressed reference genes ARF5, ERCC3 and TMEM87A using geNorm [32]. Each symbol represents the expression in an individual patient, and the horizontal line represents the median expression in the group. Statistically significant differences (*p* < 0.05; Mann-Whitney test) are indicated. **B.** Protein lysates from CD4+ T-cells isolated from peripheral blood from eight SS patients and four healthy controls were analyzed with Western blot for the presence of EPHA4 protein and EPHA4 phosphorylated at Y779 in the kinase domain (pEPHA4-Y779). The EPHA4 expression was compared to that in PC3 cells untreated (untr.) or treated with pervandate (perv.). β-actin served as loading control. **C.** Protein lysates of the SS cell lines SeAx and HuT-78 were subjected to Western blot analysis using EPHA4 antibody and expression was compared to that in PC3 cells. β-actin served a loading control.

We previously demonstrated that *EPHA4* mRNA is highly expressed in primary CD4+ cells of SS patients [11]. Here we extended this finding by investigating *EPHA4* mRNA expression in SS cells of five new SS patients and compared that to four healthy controls and four BE samples (Figure 1A). We indeed could confirm that *EPHA4* was significantly higher expressed in SS cells compared to healthy controls (*p* = 0.0079) and benign erythroderma (*p* = 0.0079). Interestingly, *EPHA4* mRNA was not expressed in the two well known SS cell lines, HuT-78 and SeAx (Figure 1A).

To investigate if high *EPHA4* mRNA expression was accompanied by increased protein expression, we performed Western blot analyses on eight primary SS samples and compared them to CD4+ T-cells from four healthy controls for the presence of EPHA4 protein (Figure 1B). EPHA4 protein could be detected in both SS samples and healthy controls. However the amount of EPHA4 protein in the primary SS cells was higher than in the CD+ T-cells from healthy controls. Consistent with the absence of mRNA, no EPHA4 protein expression was detected in the SS cell lines (Figure 1C). Unfortunately, EPHA4 protein expression could not be evaluated by immunohistochemistry in SS skin biopsies, since the EPHA4 antibody used in the Western Blot experiments was not suited for immunohistochemistry.

### The kinase domain of EPHA4 is not phosphorylated in CD4+ T-cells in SS patients

EPHA4 is activated upon binding of its ligand by subsequent phosphorylation of the tyrosine residues in the juxtamembrane segment (Y596 and Y602) and kinase domain (Y779), where phosphorylation of the kinase domain is essential for full kinase activity [34]. To assay whether the overexpressed EPHA4 in SS cells is activated, Western blot analyses was performed using a specific antibody against a phosphorylated Y779 in the EPHA4 kinase domain (EPHA4-pY779). The specificity of this antibody was verified by demonstrating detection of both commercial and in-house designed phosphorylated purified EPHA4 kinase domains [35, 36] (suppl. figure 1). None of the eight SS samples demonstrated phosphorylation of the kinase domain. In contrast, PC3 cells treated with pervanadate, a general phosphatase inhibitor, showed clear phosphorylation of EPHA4 (Figure 1B).

**Figure 2:**
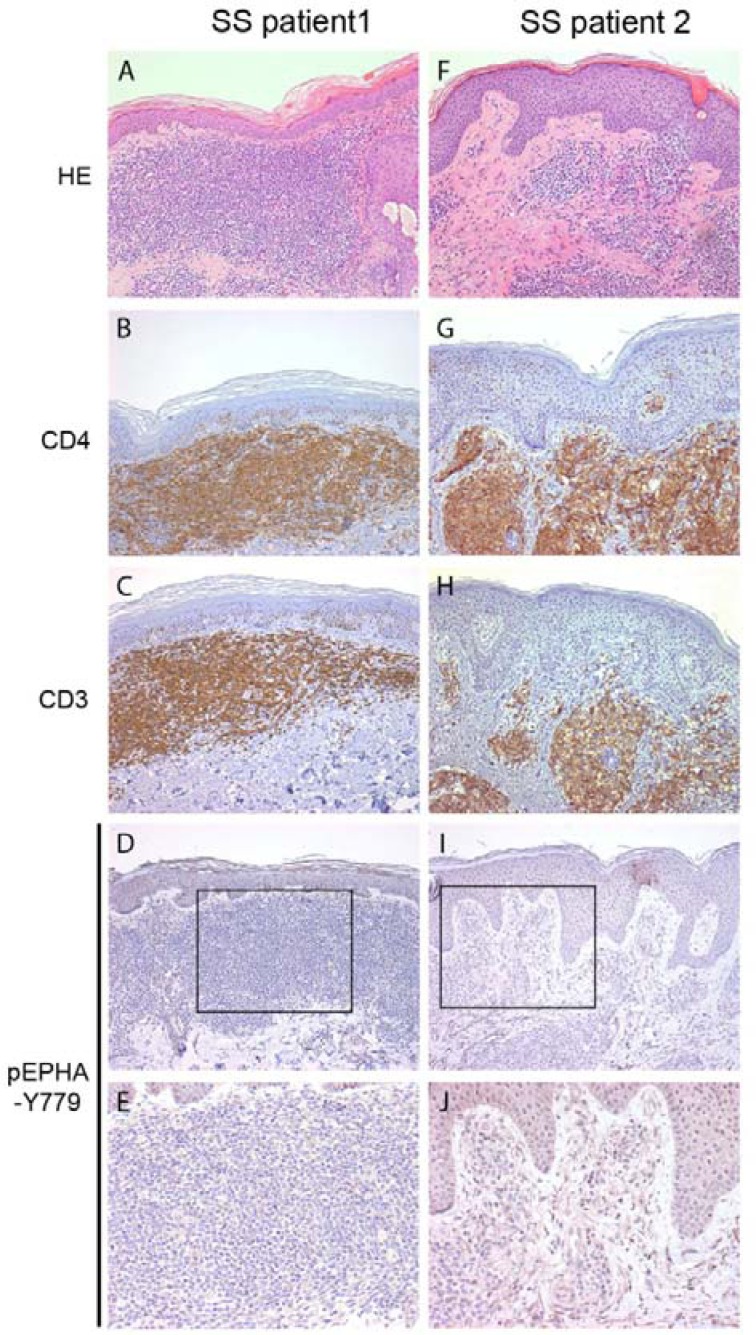
Phosphorylated EPHA4 expression in skin biopsies of SS patients Skin biopsies from SS patients (*n* = 5) were stained with hematoxylin and eosin (HE; **A.**, **F.**), and with antibodies against CD4 **B.**, **G.**, CD3 **C.**, **H.** and phosphorylated EPHA4 (pEPHA4-Y779; **D.**, **E.**, **I.** and **J.**). Representative examples are shown. In none of the biopsies clear pEPHA-Y779 positive SS cells were observed.

We hypothesized that EPHA4 could be inactive in circulating SS cells due to little cell-cell contact, but could be activated in skin where increased cell-cell contact with other cells, such as keratinocytes and fibroblasts, is likely. We therefore investigated if phosphorylated EPHA4 was detectable in six skin biopsies of SS patients using immunohistochemistry. Breast and prostate carcinoma, previously described as being positive for pEPHA4 [23, 25] were used as positive controls (results not shown), confirming the suitability of the pEHPA4-Y779 antibody for immunohistochemical analysis. In contrast, no clear positive staining was detected in CD4+ SS cells in SS skin biopsies (Figure 2A–2J). Thus, both peripheral blood and skin showed absence of phosphorylation of the kinase domain of EPHA4 in primary SS cells.

### The EPHA4 kinase domain is resilient to phosphorylation on Y779 in CD4+ T-cells from SS patients and healthy controls

**Figure 3:**
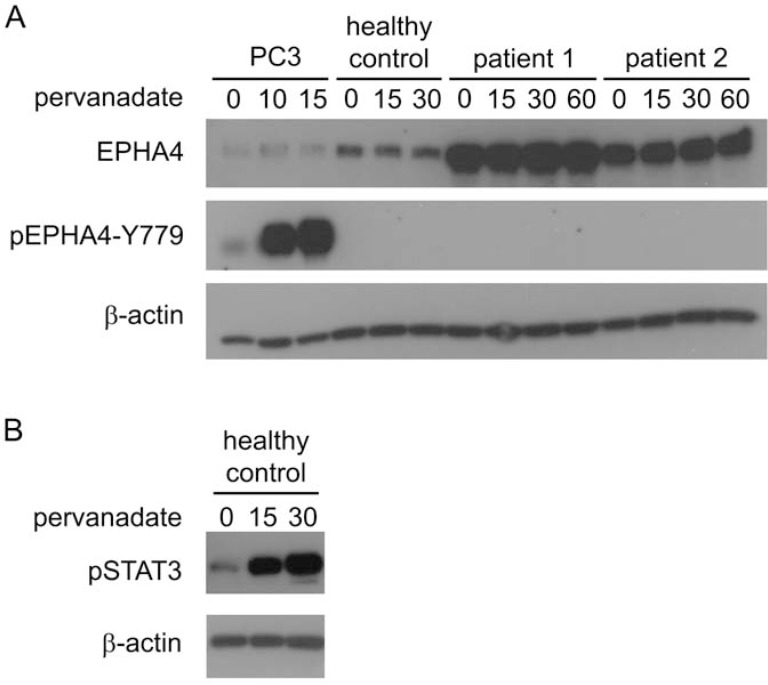
Activation of EPHA4 in CD4+ T-cells *in vitro* Cells were treated with pervanadate for the indicated time points (min) and were lysed for subsequent protein analysis with Western blot analysis. **A.** Expression of EPHA4 and phosphorylated EPHA4 (pEPHA4-Y779) in PC3 cells, and CD4+ T-cells isolated from peripheral blood from one healthy control and two SS patients. **B.** phosphorylated STAT3 (pSTAT3) in CD4+ T-cells isolated from peripheral blood from one healthy.

To investigate whether EPHA4 could be activated *in vitro*, SS cells of two patients were treated with the phosphatase inhibitor pervanadate and phosphorylation of Y779 was assayed over time (Figure 3). The effectiveness of pervanadate treatment of SS cells was confirmed by demonstrating rapid phosphorylation of STAT3. In contrast, phosphorylation of the EPHA4 kinase domain was not detectable after pervanadate treatment, even after 60 min of incubation. The experiment was repeated using CD4+ T-cells from a healthy donor, but again phosphorylation of pEPHA4-Y779 was not observed. In contrast, in PC3 cells clear phosphorylation was seen after pervanadate treatment (Figure 1B).

## DISCUSSION

In this study we were able to demonstrate overexpression of EPHA4 protein in primary CD4+ T-cells of SS patients compared to healthy controls. However, EPHA4 is not phosphorylated on its kinase domain, suggesting that EPHA4 kinase activity is not upregulated in these tumors.

Previously, genome-wide mRNA expression profiling studies by us and others have demonstrated that *EPHA4* is highly upregulated in SS cells compared to other cutaneous lymphomas, such as mycosis fungoides (MF), benign forms of erythroderma and healthy controls [11–13]. In this study, we were able to correlate the high mRNA expression to high EPHA4 protein expression in SS cells compared to healthy CD4+ T-cells.

Holen et al. demonstrated that *EPHA4* is expressed in CD4+ T-cells, more specifically in CD4+CD45RO+ memory T-cells [37]. As SS cells are skin-homing memory CD4+ CD45RO+ T-cells, the high *EPHA4* expression in SS cells is in line with the higher numbers of CD45RO+ cells in SS patients compared to healthy controls. However, the expression of CD45RO+ by SS cells can vary and is also seen in neoplastic T cells in MF [38], which have low *EPHA4* mRNA expression [11]. Thus the high expression of *EPHA4* appears to be specific for SS cells. Since all SS samples tested demonstrated high EPHA4 expression at protein level, this could serve as a novel marker for the diagnosis of Sézary syndrome. An ongoing diagnostic marker study is currently investigating the diagnostic value of potential SS markers, including *EPHA4* mRNA expression.

We also investigated the activity of EPHA4 in SS cells by looking at the phosphorylation status of a critical tyrosine residue in the kinase domain of EPHA4. The catalytic and biological activities of EPH receptors (including EPHA4) are controlled by autophosphorylation on multiple residues, which include two tyrosines within a highly conserved juxtamembrane segment (JMS) and one in the kinase domain. It is known that the tyrosine in the kinase domain of an EPH receptor needs to be phosphorylated to have a fully active kinase function [34]. For EPHA4 this tyrosine is located at position 779 (Y779). None of the peripheral blood CD4+ T-cells from SS patients or healthy controls showed phosphorylation of Y779 in the kinase domain of EPHA4. We hypothesized that absence of phosphorylated Y779 might be due to the lack of cell-cell contact in the peripheral blood and that activation might only occur when the tumor cells are present in the skin. However, no phosphorylatton of pEPHA4-Y779 was seen in CD4+ cells in SS skin samples to substantiate this hypothesis.

Strikingly, we found that *in vitro* treatment of SS cells or healthy CD4+ T-cells with a general phosphatase inhibitor (pervanadate) could not induce phosphorylation of the EPHA4 kinase domain at Y779, whereas phosphorylation of another kinase, STAT3, was effectively induced in these cells. In contrast, pervanadate treatment of PC3 cells resulted in time dependent phosphorylation of the EPHA4 kinase domain at Y779. We analyzed the entire EPHA4 coding sequence, including the region encoding Y779, in primary SS cells of three SS patients for (inactivating) mutations, but no mutations were detected (data not shown). Other studies have demonstrated phosphorylation of EPHA4 in CD4+ T-cells after treatment with soluble ligands and identified several possible downstream targets [37, 39]. The difference in outcome between their and our study may relate to the fact that we specifically assayed phosphorylation of Y779 in the kinase domain of EPHA4, whereas others determine phosphorylation of all tyrosine residues. We therefore hypothesize that stimulation of CD4+ T-cells results in EPHA4 phosphorylation outside the kinase domain, which potentially results in a kinase without full activity [34]. The activation of downstream targets may be mediated via incomplete activation of EPHA4, or via cross activation of other (EPH) receptors. The fact that downstream targets were activated might suggest that partial activation is sufficient [37]. However, whether downstream target activation was mediated *via* EPHA4, or *via* cross activation by other (EPH) receptors cannot be determined. Therefore, further studies (e.g. using selective EPHA4 inhibitors) have to be conducted to investigate this phenomenon in more detail.

Since we have now established high expression of EPHA4, most likely without accompanying kinase activity, one can speculate whether upregulation of EPHA4 is a driver of SS development or merely a by-stander effect of other oncogenic changes. EPHA4 has several transcription factor binding sites for STAT3 in its promoter, a transcription factor known to be constitutively activated in SS [8, 40]. In addition, the transcription factor TWIST1 is highly expressed in SS [11, 41], and EphA4 was shown to be a down-stream effector for Twist1 in neural development in mice [42]. Taken together, these results suggest that enhanced expression of EPHA4 is most likely a bystander effect and the consequence of highly active transcription factors as TWIST and STAT3 and is merely a by-product of oncogenic transformation. However, alternative, possible kinase-independent functions of EPHA4 cannot be excluded.

In summary, we were able to demonstrate high EPHA4 protein expression in circulating SS cells, without phosphorylation of the kinase domain. The lack of phosphorylation suggests that EPHA4 is not functionally active in SS, at least not through classical kinase receptor signaling. Therefore it might not be an attractive therapeutic target for the treatment of SS as it is in other, mostly solid, tumors using small molecule inhibitors. Instead it could be used as a diagnostic marker to distinguish SS from other CTCLs or as a target for (monoclonal) antibodies. Further studies are required to investigate this and explore whether EPHA4 has other, kinase-independent functions or whether its upregulation is merely a result of the highly active transcription factors STAT3 and TWIST1.

## MATERIALS AND METHODS

### Patient material

SS patients (three males and five females, median age 68 years) were diagnosed on the basis of criteria of the World Health Organization (WHO)/European Organization for Research and Treatment of Cancer (EORTC) classification [2–4]. Patients with benign erythroderma (BE) secondary to psoriasis (*n* = 2) and atopic dermatitis (*n* = 2) and healthy donors (*n* = 4) were included as control groups. Formalin-fixed, paraffin-embedded (FFPE) skin biopsies from six SS patients and positive controls for immunohistochemistry (breast carcinoma and prostate carcinoma) were retrieved from the pathology archives of the Leiden University Medical Center.

Approval for these studies was obtained from the Leiden University Medical Center review board, and informed consent was provided according to the Declaration of Helsinki Principles. All samples were handled in a coded fashion, following the medical ethical guidelines described in the Code ‘Proper Secondary Use of Human Tissue’ established by the Dutch Federation of Medical Sciences.

### Antibodies

In this study the following antibodies were used: Rabbit-anti-EPHA4 (ECM Biosciences, Versailles, KY, USA); Rabbit-anti-EPHA4-pY779 (ECM Biosciences); Rabbit-anti-beta-actin (Cell Signaling Technology, Danvers, MA, USA); Mouse-anti-CD3 (DAKO, Glostrup, Denmark), Mouse-anti-CD4 (Leica(Novo Castra), Newcastle Upon Tyne, UK); Goat-anti-mouse Ig-HRP (Pierce Scientific, Rockford, IL, USA); Goat-anti-rabbit Ig-HRP (Pierce Scientific); Goat-anti-rabbit Ig-biotin (Vector, Burlingame, CA, USA); Goat-anti-mouse Ig-biotin (DAKO).

### Cell lines

The SS cell lines HuT-78 (ATCC #TIB-161) and SeAx (kindly provided by Dr. K. Kaltoft) [29] and the prostate cancer cell line PC3 (ATCC #CRL-1435), were grown in HEPES buffered RPMI 1640 medium supplemented with 2 mM L-glutamine, 10% fetal bovine serum (FCS, HyClone, Thermo Scientific Rockford, IL USA), 100 Uml^−1^ penicillin, 100 mgml^−1^ streptomycin (both from Invitrogen, Carlsbad, CA, USA). For SeAx the medium was supplemented with 200 Uml^−1^ IL-2 (PeproTech, Rocky Hill, NJ, USA). The authenticity of the cell lines was verified by short tandem repeat DNA profiling.

### CD4+ T-cell isolation and culturing

Peripheral blood mononuclear cells were isolated from heparinized blood by Ficoll density centrifugation. From the mononuclear cells, CD4 + T-cells were isolated by negative selection using magnetic beads (CD4+ T-cell isolation kit; Miltenyi Biotec, Bergisch Gladbach, Germany). Purity of the CD4+ T-cell population was generally > 85%, and > 90% of the isolated CD4+ T-cells in SS samples were SS cells, characterized by expression of a clonally rearranged T-cell receptor [30].

CD4+ T-cells were cultured in RPMI-1640 supplemented with 10% human AB serum (Greiner Bio-One, Alphen aan den Rijn, The Netherlands), 2 mM L-glutamine, 100 Uml^−1^ penicillin, 100 mgml^−1^ streptomycin, 200 Uml^−1^ IL-2, and 5 ngml^−1^ IL-7 (PeproTech).

### Quantitative real time PCR (qPCR)

RNA was isolated from CD4+ T-cells from healthy controls, patients with SS or BE and the cell lines HuT-78 and SeAx using the RNeasy mini kit (Qiagen, Hilden, Germany) and included on-column DNase treatment. From 1 μg total RNA, cDNA was synthesized using the iScript^TM^ cDNA Synthesis Kit (Bio-Rad, Veenendaal, the Netherlands) according to manufacturer's instructions.

QPCR for *EPHA4* mRNA and the reference genes (*ARF5*, *ERCC3* and *TMEM87A*) with previous published primers [11, 31] were performed using the SYBR Green Supermix (Bio-Rad) on the CFX384™ real-time PCR detection system (Bio-Rad). The PCR-program included initialization (6 min at 95°C), 45 cycles of denaturation (15s at 95°C), annealing (30s at 60°C) and elongation (30s at 72°C), final elongation for 1 min at 72°C and a DNA melting curve (55°C to 95°C through 0.2°C increments every 10s). All samples were tested in duplicate.

*EPHA4* expression was normalized based on the expression of the three reference genes using the normalization factor from geNorm [32]. The difference in *EPHA4* expression between SS samples and the two control groups was analyzed with the Mann-Whitney test.

### Pervanadate treatment

Pervanadate (100 mM) was freshly made from vanadate and hydroxyperoxide (both Sigma-Aldrich, St. Louis, MO, USA) in 2 mM HEPES buffer pH = 7.3 as previously described [33]. The pervanadate was added to medium of the cells in 10mM final concentration. Cells were treated for the indicated time points before collection and subsequent lyses.

### Western blot

Cells were lysed in M-PER supplemented with Halt protease inhibitor cocktail and phosphatase inhibitor cocktail (Thermo Scientific) using 23G needle and syringe and incubated on ice for at least 15 min. The lysates were cleared by centrifugation (18×10^3^ x *g* for 20 min at 4°C). Protein concentration was determined by BCA^TM^ Protein assay (Pierce Scientific) and lysates were stored at −80°C.

Prior to separation by SDS-PAGE on a 8% gel, 7.5 μg of total protein lysate was reduced in loading buffer containing dithiothreitol by boiling at 100°C for 5 min. After transfer to methanol-activated polyvinylidene fluoride (PVDF) transfer membrane (Thermo Scientific) and blocking in 10% not-fat dry milk in Tris buffered saline with 0.1% Tween-20, membranes were incubated overnight at 4°C under agitation with the primary antibodies. Subsequently the membranes were incubated with the appropriate HRP-linked secondary antibody for 1h at RT under agitation. Immunoblots were visualized by ECL (SuperSignal West Femto Maximum Sensitivity Substrate (Pierce Scientific)) and recorded on X-ray film.

### Immunohistochemistry

Immunohistochemical staining was performed on 4μm sections of FFPE samples using standard procedures. Antigen retrieval was performed by boiling for 10 min in 10mM citrate buffer (pH = 6.0) or in Tris (10 mM)/EDTA (1 mM) (pH = 9.0) for phosphorylated EPHA4. Sections were incubated overnight at 4°C with the primary antibodies, followed by incubation with the appropriate biotinylated secondary antibody. The immunolabeling was detected by an streptavidin-biotin complex (GE Healthcare, Amersham, UK) and 3,3′-diaminobenzidine (Sigma-Aldrich) as a chromogen. Sections were counterstained with hematoxylin.

## SUPPLEMENTARY MATERIAL FIGURES


