# Supplementary material for: EPHA4 is overexpressed but not functionally active in Sézary syndrome
Source: Oncotarget. 2015 Sep 10;6(31):31868–76. doi: 10.18632/oncotarget.5573 (PMC4741646; doi:10.18632/oncotarget.5573)
Supplement: Supplementary file 1 [file oncotarget-06-31868-s001.pdf]

EPHA4 is overexpressed but not functionally active in Sézary syndrome

Supplementary Material

Supplementary figure 1

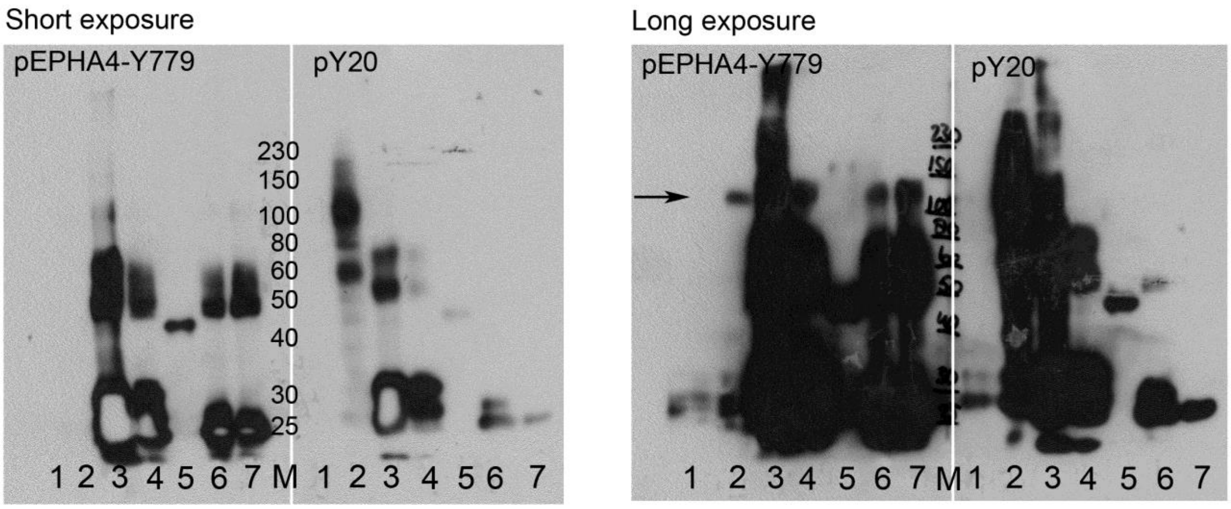

1. PC3 perv t=0
2. PC3 perv t=15min
3. EPHA4-KD-his (37kDa)
4. EPHA4-KD-his (37kDa)
5. EPHA4-KD-GST (60kDa)
6. EPHA4-KD (32kDa)
7. EPHA4-KD (32kDa)

M. Size marker (kDa)

Supplementary figure 1. Phosphorylation of EPHA4 kinase domain in EPHA4 constructs demonstrating the specificity of the pEPHA4-Y779 antibody.

Western blot analysis of different phosphorylated purified EPHA4 kinase domain constructs, together with PC3 cells with or without pervanadate treatment to induce phosphorylation. The blots were probed with pEPHA4-Y779 or pY20 antibodies to detect phoshorylation of EPHA4 KD and total phosphorylation of tyrosines respectively (short and long exposure).

The commercially available GST-tagged protein (EPHA4-KD-GST, lane 5) nicely shows the presence of a phosphorylated protein both with the pY20 antibody and the pEPHA4-Y779 antibody. In addition, two different home-made protein constructs were tested, a his-tagged KD (EPHA4-KD-his, lanes 3 and 4) and an untagged KD (EPHA4-KD, lanes 6 and 7). Details of the construction, production, purification and phosphorylation of the home-made protein is described elsewhere

[1,2]. For both constructs, reactivity with the pY20 antibody and the pEPHA4-Y779 antibody was observed. Untreated PC3 cells did not show any reactivity with the pEHPA4-Y779 or the pY20 antibody. Multiple phosphorylated proteins were detected in the pervanadate-treated PC3 cells with the pY20 antibody, whereas a specific single band was observed with the pEPHA4-Y779 antibody (indicated with an arrow). Taken together these results confirm the specificity of the pEPHA4-Y779 antibody.

Reference List

1. Farenc C, Celie PH, Tensen CP, de Esch IJ and Siegal G (2011) Crystal structure of the EphA4 protein tyrosine kinase domain in the apo- and dasatinib-bound state. FEBS Lett. 585:3593-3599

2. van Linden OP, Farenc C, Zoutman WH, Hameetman L, Wijtmans M, Leurs R, Tensen CP, Siegal G and de Esch IJ (2012) Fragment based lead discovery of small molecule inhibitors for the EPHA4 receptor tyrosine kinase. Eur. J. Med. Chem. 47:493-500
